# Inbreeding and selection on sex ratio in the bark beetle *Xylosandrus germanus*

**DOI:** 10.1186/1471-2148-11-359

**Published:** 2011-12-13

**Authors:** Laurent Keller, Katharina Peer, Christian Bernasconi, Michael Taborsky, David M Shuker

**Affiliations:** 1 Department of Ecology and Evolution, University of Lausanne, Biophore, CH 1015 Lausanne, Switzerland; 2 Behavioural Ecology, Institute of Ecology and Evolution, University of Bern, Wohlenstrasse 50a, CH- 3032 Hinterkappelen, Switzerland; 3 Trinserstrasse 31, A-6150 Steinach, Austria; 4 School of Biology, Harold Mitchell Building, University of St Andrews, St Andrews, KY16 9TH, UK

## Abstract

**Background:**

Local Mate Competition (LMC) theory predicts a female should produce a more female-biased sex ratio if her sons compete with each other for mates. Because it provides quantitative predictions that can be experimentally tested, LMC is a textbook example of the predictive power of evolutionary theory. A limitation of many earlier studies in the field is that the population structure and mating system of the studied species are often estimated only indirectly. Here we use microsatellites to characterize the levels of inbreeding of the bark beetle *Xylosandrus germanus*, a species where the level of LMC is expected to be high.

**Results:**

For three populations studied, genetic variation for our genetic markers was very low, indicative of an extremely high level of inbreeding (F_IS _= 0.88). There was also strong linkage disequilibrium between microsatellite loci and a very strong genetic differentiation between populations. The data suggest that matings among non-siblings are very rare (3%), although sex ratios from *X*. *germanus *in both the field and the laboratory have suggested more matings between non-sibs, and so less intense LMC.

**Conclusions:**

Our results confirm that caution is needed when inferring mating systems from sex ratio data, especially when a lack of biological detail means the use of overly simple forms of the model of interest.

## Background

To avoid the cost associated with inbreeding, many organisms have evolved behavioural adaptations preventing matings between close relatives [1-7]. However, in some species, matings commonly occur between kin, the most striking examples being species in which the offspring produced by a female are restricted to mate amongst themselves, for instance before mated females disperse to find new resource patches [8-10]. When kin compete locally with each other for mates, W.D. Hamilton showed that biased sex ratios are favoured by a process he termed Local Mate Competition (LMC; [11]). Under LMC, females are selected to produce less of the sex that competes for mates, typically males, leading to female-biased sex ratios. In the extreme case of strict local mating and a single breeding female per patch, the model predicts that the reproductive female should produce only the minimum number of sons needed to fertilize all her daughters. Moreover, when the extent of LMC changes (for instance if broods from unrelated females interact, reducing competition amongst brothers), females are selected to alter their sex ratios in a facultative fashion. With an increasing number of females reproducing within one patch, the optimal sex ratio becomes less female biased and is predicted to approach equality [12].

Because it provides easily testable quantitative predictions, LMC theory is a textbook example of the predictive power of evolutionary theory [8,9,13-20]. The most extreme cases of local mate competition and female-biased sex ratio are found in haplodiploid insects such as ambrosia beetles, parasitoid wasps and fig wasps [9,21-24]. In haplodiploid species, optimal sex ratio further depends on the level of inbreeding, because inbreeding increases the relatedness asymmetry between a mother and her sons and daughters. Females are always equally related to their sons, but with increasing levels of inbreeding they become more related to their daughters. The optimal sex ratio is therefore expected to become slightly more female-biased with higher levels of inbreeding [9,14,25].

Although predictions of LMC theory have been tested across a range of organisms [20], one major problem is that the population structure and mating system of the species studied (i.e. the extent of LMC among related males) are often estimated only indirectly [21,26,27]. Accordingly, several assumptions need to be fulfilled for these estimates to yield reliable predictions of sex ratio under LMC. For example, the most straightforward LMC models assume that when several females reproduce together on a patch, all of them produce the same size of broods, the same sex ratio and that their offspring should mate randomly within the patch. However, in truth all these assumptions will typically be violated, especially as females may contribute eggs or young to a resource patch sequentially, such that later females are faced with a lower quality resource and therefore produce a smaller brood - a key determinant of sex ratio (e.g. [28]). Fortunately, a variety of more sophisticated models of LMC exist, developed as researchers gained insight into the biology of particular species (reviewed by [20]). For example, in a recent study in the parasitoid wasp *Nasonia vitripennis*, Burton-Chellew et al ([24]) genotyped all the broods from a number of naturally occurring patches in order to estimate foundress numbers and relative clutch sizes across each individual host within a patch. They were therefore able to test alternative models of LMC that allow each of these variables to vary, and showed that an LMC model based on relative clutch size at the individual host level was the best predictor of sex ratio (see also [27]). However, the extent to which within-patch variation in LMC leads to population-wide discrepancies in predictions of sex ratio is not generally known; in *Nasonia vitripennis*, the population inbreeding coefficients did provide a reasonable estimate of the mating system of an "average patch" [21,24,27].

The aim of this study was to use microsatellites to characterize the levels of inbreeding in three populations of the bark beetle *Xylosandrus germanus*, a species that experiences strong local mate competition [23]. The Xyleborini (Ambrosia beetles, Scolytinae) are a species-rich tribe of bark beetles with haplodiploid sex determination [29,30]. Probably all xyleborine species show high levels of inbreeding through regular sib mating, and offspring sex ratios are strongly female-biased [29,31,32]. Males are flightless and short-lived, while females disperse and excavate individual breeding galleries in freshly dead trees. Daughters may delay dispersal and help in fungus care and the raising of siblings, whilst males may attempt to find matings in other galleries on the same log [29,33-37]. The mating system, brood sex ratios and the fitness consequences of the mating behaviour of *X. germanus *have been investigated in previous studies [10,23]. As with other xyleborine species, these studies initially assumed that mating occurs exclusively in the natal brood chamber and males die without dispersing since they cannot fly. However, logs may be densely colonized with over 100 galleries per log and field observations revealed that males do try to gain access to other galleries in a log [23]. As such, females have been shown to alter facultatively the sex ratio of their broods according to colonization densities (i.e., the presumed outbreeding opportunities for their sons: [23]). However, the success of these mating forays by males and the resulting rates of mating between non-sibs are still unknown. Experimental crosses within and between broods did not indicate inbreeding depression, but rather outbreeding depression [10].

Here we test whether the population genetic estimates of the inbreeding coefficient across three *X*. *germanus *populations match those predicted by the estimates of sex ratio obtained in both the laboratory and the field, in order to better understand the mating system in the wild. If within-patch LMC varies rather little (or averages out across patches) and females disperse randomly from their natal patches, our estimates of the inbreeding coefficient should predict sex ratios of between 0.06 and 0.10 [23]. However, if population sub-structuring is influenced by factors beyond sib-mating on the natal patch (such as female dispersal behaviour) our inbreeding coefficients will provide less robust estimates of the actual mating system of *X*. *germanus*. Our results show extremely limited genetic variation across eight microsatellite marker loci, indicating higher levels of inbreeding than that predicted by observed sex ratios.

## Results

Six of the eight loci tested were completely monomorphic in the three populations and were excluded from further analyses. Only Cdact2 and Ccarp15 were polymorphic (Table 1). Overall, we genotyped 495 individuals at these 2 loci. The number of alleles was very low with only two alleles scored for both Cdact2 and Ccarp15. All the individuals genotyped in populations B and M were homozygous and had identical genotypes at all loci. Only population S showed some polymorphism: the two alleles for both loci were represented, but only two of the four possible genotypes were at all common (Table 2). There was thus very strong linkage disequilibrium (Δ' = 0.9) between these two loci.

**Table 1:** Allelic frequencies for each allele in each population

	Locus 1 (Cdact2)		Locus 2 (Ccarp15)	
	
	Allele A	Allele B	Allele C	Allele D
Pop Spilwald	0.614	0.386	0.596	0.404
Pop Bremgartenwald	0	1	0	1
Pop Mühleberg	0	1	0	1

**Table 2:** Frequencies of the genotypes observed within the Spilwald population

Locus 1 (Cdact2)	Locus 2 (Ccarp15)	Freq
AA	CC	0.59
AA	CD	0.03
AB	CD	0.03
BB	CC	0.01
BB	DD	0.33
BB	CD	0.01

The inbreeding coefficient within population S was very high (F_IS _= 0.88) and significantly greater than zero when estimated over all loci (p < 0.05). F_IS _values for single loci were significant for both Cdact2 (F_IS _= 0.94, p < 0.05) and Ccarp15 (F_IS _= 0.81, p < 0.05; jack-knifing was not possible because of the small number of polymorphic loci). The F_ST _value over the three populations was also very high (F_ST _= 0.73, p < 0.05), indicating very limited gene flow across forests (again, jack-knifing was not possible because of the small number of polymorphic loci).

The estimated proportion of sib-matings was higher than that predicted under simple assumptions of inbreeding associated with LMC among related males, with sib-mating appearing practically ubiquitous. The estimated proportion of sib-matings from the F_IS _value (0.88) was 0.97. The predicted proportion of sib-matings was lower when estimated from the sex ratios obtained in the field (r = 0.063) and laboratory (r = 0.1; [23]), which gave estimated rates of sib-mating of s_f _= 0.75 and s_l _= 0.83, respectively.

## Discussion

As with some other studies of bark beetles, genetic polymorphism across the three populations of *X*. *germanus *was limited (e.g. [38-40]). The genetic analyses revealed an extremely high level of inbreeding as well as a very strong genetic differentiation between populations of *X. germanus *(see also Ito et al [41] for study of Japanese populations of *X*. *germanus*). The level of inbreeding in the Spilwald forest (the only population exhibiting variability at the markers considered) suggests that 97% of all matings occur among siblings. As such, we might have expected even more extreme female-biased sex ratios than those observed thus far in the laboratory and in the wild. Those latter data suggested sib-mating rates of between 75% and 83% (from field and lab estimates of sex ratio, respectively [23]; see [36] for a similar result in *Xyleborinus saxesenii *lab galleries). Our data suggest that intense local mate competition (LMC) is common, which fits with the biology of the Xyleborini. In *X. germanus*, a single foundress female excavates the brood chamber herself and blocks the entrance tunnel against any intruders [31,42]. Thus, all broods result from a single foundress [23], leading to the assumption of strict sib-mating within the natal gallery. However, field observations have suggested that a small proportion of males successfully disperse to other galleries within the log, consistent with the finding that females increase their brood sex ratios at higher colonization densities according to LMC theory [23]. Despite this, the results presented here suggest that the proportion of dispersing males that actually manage to inseminate non-sibs is very small.

To what extent might the differences between the observed and expected levels of inbreeding be real or artefactual? There are several possible explanations for the discrepancy between the previously collected sex ratio data and the genetic analysis. The first is sampling bias of the field sex ratios. More densely colonized logs are easier to detect and thus more likely to be collected. Experiments with different colonization densities on artificial medium in the laboratory revealed that females adjust the sex ratio to the potential outbreeding opportunities of their sons, producing more males at higher colonization densities [23]. This facultative sex allocation will bias our impression of *X*. *germanus *sex ratios if we have previously only collected sex ratio data from lower LMC (higher density) logs. If high LMC (single foundress) logs are more common than we have thus far sampled, population sex ratios may be more female biased, which might reconcile the sex ratio and inbreeding data to some extent.

Second, female dispersal may be non-random at the population level, such that sub-structuring occurs because of both sib-mating on patches, and non-random dispersal among patches by mated females (e.g. they disperse to logs close to their natal patch, such that co-foundresses may be more related than expected by chance). Indeed, differential dispersal of males and females can influence LMC and female-biased sex ratios independently of sib-mating (as has been suggested to be the case in the wasp *Habrobracon hebetor *[43]). Fortunately, models of LMC can be built that allow relatedness among individuals to be influenced by both sib-mating within patches and non-random association of foundresses (e.g. [44]), although typically this added layer of complexity is ignored. In the parasitoid wasp *Nasonia vitripennis*, females do not use local relatedness of foundresses as a cue for sex ratio [44-46]. How *X*. *germanus *respond to the relatedness of co-foundresses is not yet known, but two lines of evidence suggest that there is perhaps limited or non-random dispersal among patches. First, there was a very high genetic differentiation between the three populations studied as revealed by the high F_ST _value, even though the forests were only 5-10 kilometres apart (this might be within the potential dispersal range of females; cf. [41]). Such a pattern of dispersal could explain both the low within-population diversity and the high between-population differentiation. Indeed, six of eight of the microsatellites analysed were completely monomorphic in all populations and the two others also exhibited a complete lack of genetic variability in two of the three populations (Bremgartenwald and Mühleberg). Finally, the combination of high inbreeding levels and limited dispersal may also account for the observed very strong linkage disequilibrium between alleles at the two polymorphic loci in the only population exhibiting any level of genetic variability (Spilwald). The unusual dispersal pattern of *X*. *germanus *might also account for the level of inbreeding being above the value predicted by sex ratio models, especially if females do not vary their sex ratios in response to the relatedness of co-foundress females. *X*. *germanus *may therefore be a good candidate for a more detailed analysis combining biologically explicit LMC models with sex ratio and inbreeding data from a greater number of patches and populations.

Third, *X*. *germanus *has only recently been introduced into Europe from East Asia [47,48]. This means it is possible that any changes in the breeding system and patterns of dispersal associated with this introduction are not yet in equilibrium with the observed sex ratio pattern. As sex ratios close to the optimum are typically under weak selection, if may take a number of generations for sex ratios to reflect precisely the new selective regime (i.e. the level of inbreeding and degree of LMC). Apparently *X*. *germanus *has been introduced to Switzerland only 15 years before they were collected for this study [49] and it is unknown whether there are differences in the breeding systems of the populations we studied with those of the native habitat.

Fourth, there may be constraints on the extent to which sex ratios can be reduced, remembering that single foundresses are expected to produce the minimum number of males necessary to fertilise all their daughters. If male mortality at the larval, pupal and early adult stages is an important hazard, mothers may produce more males than otherwise expected in order to insure fertilization of their daughters' eggs (e.g. [23,50,51]). Similarly, in the malaria parasite *Plasmodium*, that also experiences sex ratio selection in the form of LMC, male gametocytes appear to be over-produced to avoid sperm-limitation [52-54]. More prosaically, if clutch sizes are small, integer effects come into play, such that sex ratios are constrained from becoming too female biased (for instance if at least two males are always needed, single digit clutch sizes will never be extremely female-biased: [16,55]).

Finally, is there a role for any other form of sex ratio selection? Interestingly, recent data have suggested that female offspring in the related species of *Xyleborinus saxesenii *sometimes remain in the natal gallery and appear to help raise further brood (i.e. their presence improves colony productivity: [34,35,37]). Cooperative brood care has been observed also in *X. germanus *[33]. Cooperative breeding with help coming from the offspring of one sex can select for biased sex ratios that favour production of the helping sex (in this case females) by a process called Local Resource Enhancement (or LRE; [56,57]). If the same applies in *X*. *germanus*, then female biased sex ratios may result from a combination of both LMC and LRE.

## Conclusions

This study suggests that the combination of limited dispersal and frequent mating between sibs leads to a very high level of inbreeding in *X. germanus*. The observed level of inbreeding was higher than that expected from the sex ratio data under the assumptions of a simple LMC model, which confirms that inferences made on the mating system from sex ratio data should be treated with care [22]. Without a proper understanding of the biology of a focal species, attempts to use general sex ratio theory may therefore fail. In the present case, alternative selection pressures may influence sex ratio (including the patterns of female dispersal, fertilization insurance and LRE), and appropriate models and field data will therefore be needed to test sex allocation theory in *X*. *germanus *in the wild, as in other species.

## Methods

### Sampling

Sampling was conducted in 2001 and 2002 in forests surrounding Bern, Switzerland. Beetles were sampled in three forests: Bremgartenwald (B), Spilwald (S) and Mühleberg (M). These forests are separated by about 5-10 kilometres from each other. Because dispersing females use ethanol to locate suitable host trees, we set up ethanol-baited live traps to capture them. Traps were placed at 7 locations in Bremgartenwald (4 traps in 2001, 4 traps in 2002), 9 locations in Spilwald (8 traps in 2001, 5 traps in 2002) and 4 locations in Mühleberg (4 traps in 2001, 2 traps in 2002). The trap locations within the same forest were separated by between 100 and 500 metres. Traps were checked once per week throughout the season and all specimens were stored in 95% ethanol until DNA extraction.

### Genetic analysis

We tested eight microsatellite loci (Cdact2, Cdact3, Cdact4, Cdact6, Cdact10, Ccarp2, Ccarp11 and Ccarp15 [38]). These loci were among a number developed for two species of *Coccotrypes *bark beetles, and the number of alleles for these loci in these two species varied from 1 to 5 [38]. Moreover, a recent population genetic study using six of these loci across 59 populations of *Coccotrypes dactyliperda *found variation in the number of polymorphic loci (from 0 to 6; [39]), suggesting that polymorphism may be limited in these typically rather inbred insects. Genomic DNA was isolated from 495 individuals (347 from B, 80 from S and 68 from M) using a Chelex solution (5%). PCR conditions were as described by [38] but some optimizations for *X*. *germanus *were necessary (C. Bernasconi and K. Peer unpublished). Primers were labelled with HEX, NED and FAM fluorescent dyes and the amplification products were analyzed on an ABI Prism 377 XL DNA sequencer (Applied Biosystems, Foster City, CA). Alleles were scored by length and genotyping was carried out using the computer program Genescan 3.02 (Perkin Elmer ABI).

### Estimation of sibmating levels

The observed levels of inbreeding were compared to those expected from the proportion of sibmating estimated from previously collected sex ratio data. Assuming a simple model of LMC, the proportion of sibmating(s) can be estimated (i) from the observed sex ratio (r) as follows: s = [3 - r - (1 + 10r + r^2^)^0.5^]/2 [58,59] or (ii) from the inbreeding coefficient F as follows: s = 4F/(1 +3F) [26].

### Genetic structure

The genetic structure of the three populations was characterized by Wright's fixation indices [60,61]. Standard errors of F-statistics were obtained by jack-knifing and confidence intervals were obtained by permutation tests over loci (10,000 permutations; [62]). Traps were treated as subunits of the same population. Linkage disequilibrium was calculated in order to estimate the amount of recombination between alleles. Calculations were done by FSTAT ver. 2.9.2 [62].

## Authors' contributions

LK, KP, CB, MT conceived the study, and collected and analysed the data, and contributed to the writing of the paper. DS contributed to the writing of the paper. All authors read and approved the final manuscript.
